# Digital Clinical Communication for Families and Caregivers of Children or Young People With Short- or Long-Term Conditions: Rapid Review

**DOI:** 10.2196/jmir.7999

**Published:** 2018-01-05

**Authors:** Xavier Armoiry, Jackie Sturt, Emma Elizabeth Phelps, Clare-Louise Walker, Rachel Court, Frances Taggart, Paul Sutcliffe, Frances Griffiths, Helen Atherton

**Affiliations:** ^1^ Warwick Medical School University of Warwick Coventry United Kingdom; ^2^ Florence Nightingale Faculty of Nursing, Midwifery & Palliative Care Kings College London London United Kingdom; ^3^ Centre for Health Policy University of the Witwatersrand Johannesburg South Africa

**Keywords:** digital clinical communication, professional-family relations, family, caregivers, young adult, children, child health

## Abstract

**Background:**

The communication relationship between parents of children or young people with health conditions and health professionals is an important part of treatment, but it is unclear how far the use of digital clinical communication tools may affect this relationship.

**Objective:**

The objective of our study was to describe, assess the feasibility of, and explore the impact of digital clinical communication between families or caregivers and health professionals.

**Methods:**

We searched the literature using 5 electronic databases. We considered all types of study design published in the English language from January 2009 to August 2015. The population of interest included families and caregivers of children and young people aged less than 26 years with any type of health condition. The intervention was any technology permitting 2-way communication.

**Results:**

We included 31 articles. The main designs were randomized controlled trials (RCTs; n=10), cross-sectional studies (n=9), pre- and postintervention uncontrolled (pre/post) studies (n=7), and qualitative interview studies (n=2); 6 had mixed-methods designs. In the majority of cases, we considered the quality rating to be fair. Many different types of health condition were represented. A breadth of digital communication tools were included: videoconferencing or videoconsultation (n=14), and Web messaging or emails (n=12). Health care professionals were mainly therapists or cognitive behavioral therapists (n=10), physicians (n=8), and nurses (n=6). Studies were very heterogeneous in terms of outcomes. Interventions were mainly evaluated using satisfaction or acceptance, or outcomes relating to feasibility. Clinical outcomes were rarely used. The RCTs showed that digital clinical communication had no impact in comparison with standard care. Uncontrolled pre/post studies showed good rates of satisfaction or acceptance. Some economic studies suggested that digital clinical communication may save costs.

**Conclusions:**

This rapid review showed an emerging body of literature on the use of digital clinical communication to improve families’ and caregivers’ involvement in the health management of children or young people. Further research with appropriate study designs and longer-term outcome measures should be encouraged.

**Trial Registration:**

PROSPERO CRD42016035467; http://www.crd.york.ac.uk/prospero/display_record.php?ID=CRD 42016 035467(Archived by WebCite at http://www.webcitation.org/6vpgZU1FU)

## Introduction

Digital clinical communication can be defined as a means of communication between a clinician and a person, when the clinician or the person (or both) is (or could be) mobile when sending or receiving the communication, in a 2-way, synchronous or asynchronous manner, and for clinical care purposes only [1]. The use of digital clinical communication technologies has been extensively described due to their capacity to facilitate communication between health care professionals and patients [2-4]. With children and young people being prolific users of these technologies, there is much speculation about the potential feasibility of digital communication between children and young people with health conditions, on the one hand, and their health professionals, on the other, as a way to meet the specific needs of this population [5]. Indeed, young people are particularly at risk of disengaging from health services and experience poorer health outcomes [1,6-8]. Although the technology is promising, the effectiveness of digital communication with patients or parents of children and young people in health care on outcomes has not been clearly demonstrated [9].

Parental involvement and parent-health professional relationships are an important part of the treatment journey of children and young people with health conditions, but it is unclear what impact digital communication has on these relationships, particularly as young people transition into using adult services. Digital communication with health care providers may also be used by families involved in the management of health conditions in much younger pediatric populations, where parents are fully acting as communicator with health services [10,11].

Given the wide spectrum of these digital tools and the different modalities used by families, we aimed to review the literature and the emerging conceptualization of the topic.

For this purpose, we chose a rapid review method, which can be defined as a form of knowledge synthesis in which components of the systematic review process are simplified or omitted to produce information in a timely manner [12]. Rapid reviews are useful in fields where change is ongoing [13], such as in the development of digital technologies.

The aim of this review was to establish the current evidence base for the use of digital clinical communication for families and caregivers of children or young people with short- or long-term conditions.

Our objectives were to describe existing digital communication use by health professionals with families or caregivers of children or young persons with short- or long-term conditions, to assess the feasibility of using these technologies, and to explore their impact on (1) family and caregivers’ outcomes, (2) children and young people’s outcomes, (3) health professionals’ outcomes, and (4) health service delivery and health economics outcomes.

## Methods

We report this review according to the Preferred Reporting Items for Systematic Reviews and Meta-Analyses (PRISMA) guidelines [14].

### Inclusion and Exclusion Criteria

Our inclusion criteria were (1) studies of any design published in the English language (except conference abstracts and articles with fewer than 5 participants); (2) family members or caregivers of individuals less than 26 years of age and presenting with all types of health condition; we extended the age range to 25 years to include literature on the issue of transition from pediatric to adult health services; and (3) all forms of 2-way digital communication between family members or caregivers and health professionals, including email; social networking sites; mobile telephony; short message service (SMS) text messaging systems; video- and teleconferencing; online forums; and electronic monitoring.

We excluded (1) technologies involving only 1-way data transmission, communication between family members or caregivers and children or young people, and communication between family members or caregivers; and (2) technologies involving 2-way communication between children or young people and professionals if there was no involvement of family members or caregivers.

In accordance with the Cochrane Consumers & Communication Group’s taxonomy [15], we were interested in studies that included patient outcomes, family and caregiver outcomes, health professional outcomes, and health service delivery and economic outcomes. We assessed feasibility according to the simple definition “the state or degree of being easily or conveniently done.” This was assessed via patient and health care professional outcomes and via reporting of technical or usability concerns. We assessed the impact of digital clinical communication only through controlled studies, while we assessed feasibility using both controlled and uncontrolled studies.

### Search Strategy

We developed a literature search strategy to search 5 electronic databases (MEDLINE [through Ovid], Embase [Ovid], MEDLINE In-Process & Other Non-Indexed Citations [Ovid], PsycINFO [ProQuest], and Cochrane Library [Wiley]) in August 2015 for relevant literature published in or after January 2009. We chose this time period to include the most recent digital communication tools. We used a combination of free-text and thesaurus terms for the concepts of technology, clinical communication, population, and families and caregivers” to identify related literature (Multimedia Appendix 1). We also searched citations and the reference lists of relevant studies. Furthermore, we hand searched within JMIR journals (themes: *Clinical Communication*; *Electronic Consultation and Telehealth*; *Email & Web-Based Communication*; *Personal Health Records*; *Patient-Accessible Electronic Health Records*; *Patient Portals*) over the same period.

### Screening and Analysis

Two independent reviewers screened all identified bibliographic records by title and abstract. We obtained full-text articles for all remaining records, and these were read by 1 reviewer. The final list of included studies at full-text level was validated by 2 other reviewers. One reviewer extracted data from all the included studies using an a priori-defined, prepiloted extraction sheet that was designed by the same reviewer and included data on the population, intervention, comparator (where relevant), and outcomes. A second independent reviewer double-checked the extracted data. A third reviewer resolved any disagreement.

We assessed the quality of randomized controlled trials (RCTs), economic evaluations, and qualitative research articles using the Critical Appraisal Skills Programme checklists [16]. For pre- and postintervention uncontrolled (pre/post) studies, cross-sectional and observational studies, and non-RCTs, we assessed study quality using checklists published by the US National Institutes of Health [17]. We used these checklists because, in the context of a rapid review, these provide a useful way to assess several different study designs consistently and quickly. For mixed-methods studies, we undertook a quality assessment for each study method. Quality was assessed independently by 2 reviewers. Any disagreement between reviewers was resolved by consensus or with recourse to a third reviewer. We rated the overall quality of studies as poor, fair, or good. This rating was assessed by the 2 reviewers through discussion, accounting for each study’s limitations as emphasized by the items within the checklists. For example, a study with high risk of bias or major flaws based on several checklist items translated to a rating of poor quality. Conversely, a study with low risk of bias or free from major flaw translated to a rating of good quality. We summarized study, intervention, population, and outcome characteristics narratively and in summary tables. Meta-analysis and statistical pooling were not possible owing to the heterogeneity of interventions and health conditions that we identified. This rapid review was registered in PROSPERO (CRD42016035467).

## Results

### Characteristics of Included Studies

**Figure 1:**
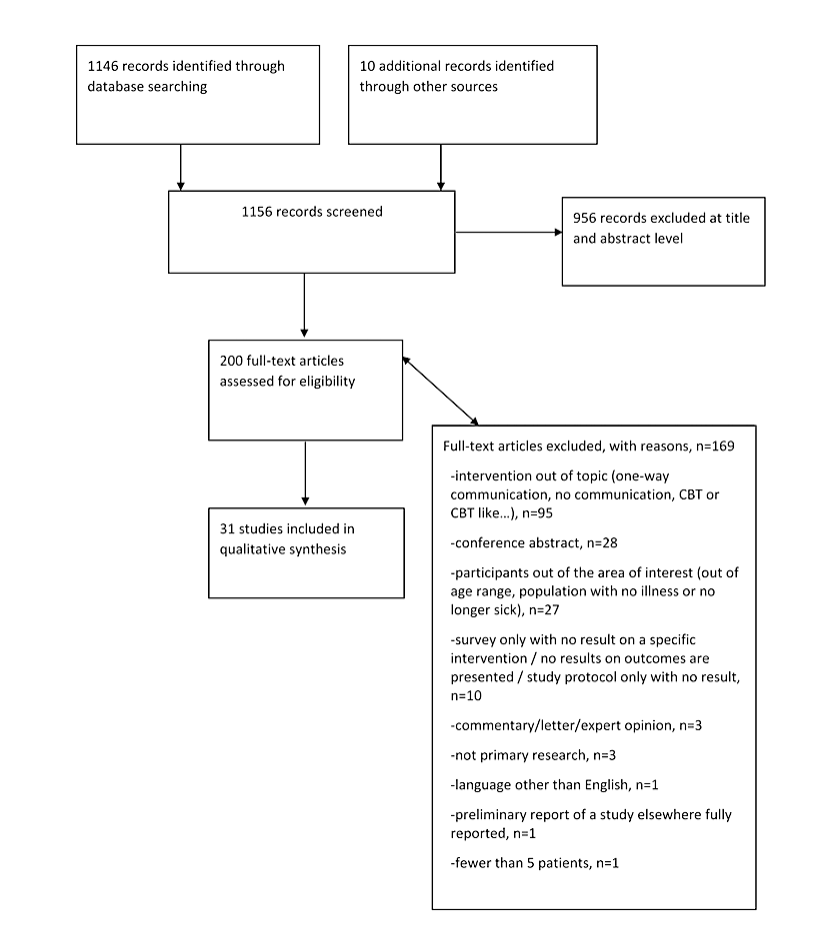
Flow diagram of study identification and analysis for inclusion. CBT: cognitive behavioral therapy.

Of the 1156 identified records (including 10 additional records from reference lists of relevant studies), we removed 956 not meeting our inclusion criteria at title and abstract stage, leaving 200 articles to be examined at full-text review. Among these, there was 1 systematic review of interest [18], which we checked for the presence of potentially relevant articles. We excluded 169 articles not meeting our inclusion criteria, leading to a total of 31 included publications (Figure 1).

The main study designs were RCTs (n=10), cross-sectional studies (n=9), pre/post studies (n=7), and qualitative interview studies (n=2) (Multimedia Appendix 2) [10,11,19-47]. Of the studies, 6 had mixed-methods designs. Most of the studies were conducted in the United States (n=17), while the other main locations were Australia (n=3), the Netherlands (n=3), and Sweden (n=3).

We identified a broad range of conditions: traumatic brain injury (n=5), the management of prematurity and associated consequences (n=3), atopic dermatitis (n=2), autism spectrum disorder (n=2), type 1 diabetes (n=2), palliative care for different types of diseases (n=2), and anorexia nervosa (n=1). The range of the mean age of children and young people was 24 days to 20.4 years. Of the 31 selected articles, 23 included a mainly pediatric population (age <12 years), while 7 mainly included adolescents (12-18 years) and 1 mainly included young adults (>18 years). Of the 7 articles including adolescents, the involvement of families and caregivers in using the digital clinical communication technology was the key component of the intervention because of the health condition of adolescents (mainly traumatic brain injury or cerebral disability).

We found no study on the use of digital communication by families or caregivers of young people during the transition to adult care.

We rated the majority of studies as being of fair quality. The main limitation was the uncertainty as to whether the participants were representative of those in the general population (for studies where generalizability was relevant). Multimedia Appendix 3 reports the full description of included studies and our quality assessment of them.

### Description of Existing Digital Communication

A range of digital communication channels were used across studies. Videoconferencing allowing consultation was predominantly used (n=14) followed by emails or Web messaging systems (n=12) (Table 1).

Health care professionals were mainly therapists or cognitive behavioral therapists (n=10), physicians (n=8), and nurses (n=6). Stand-alone interventions were used in 13 studies, while in 18 studies the digital communication was included within a wider intervention such as a Web-based therapy or Web-based system (portal, telemedicine, telehealth) that allowed 2-way communication between health care professionals and families.

### Family and Caregiver Outcomes

Multimedia Appendix 4 summarizes the results and Multimedia Appendix 5 reports the complete results. The benefits reported include removing barriers to communication [47], providing reassurance for those with chronic illness [47], and feeling supported for those adolescents with eating disorders [29]. The majority of families felt satisfied with the digital communication tools [40,41]. All the families found these tools easy to use [20,30,35,44] and some said they would recommend them [39,40].

We found no studies reporting a difference in family and caregiver outcomes between the group using digital communication and the control or alternative intervention group (eg, telephone) [10,21,26,28,36,43].

**Table 1:** Description of digital clinical communication tools as identified in the rapid review.

Digital clinical communication tool	Studies (n)
Videoconferencing systems allowing consultations	14
Web messaging or emails	12
Web chat	2
Web-based telemedicine systems with no other element	2
Short message service (SMS) text messaging	1

A total of 6 studies reported improvements for families and caregivers after the intervention compared with baseline [10,26,29,33,34,36], while 2 reported no difference [30,32]. For the parents of adolescents with eating disorders, there was an improvement on the Eating Disorders Symptom Impact Scale after participation in Web chat sessions with fellow parents and a clinical psychologist [29].

In an intervention providing early autism training to parents, the Maternal Behavior Rating Scale (designed to assess the quality of maternal interactive behavior with children with learning difficulties) score was increased compared with baseline after interventions that included videoconferencing and a telehealth-delivered curriculum [34] or Web-based learning modules [33]. Caregiver distress was reduced after the intervention [26]; the delivery of pediatric palliative home care via videoconsultation improved scores on health-related quality of life [36]. Lastly, the number of skin care treatments given by parents of children with atopic dermatitis was increased after Web-based consultations [10]. Of these 6 studies [10,26,29,33,34,36], 3 [10,26,36] found similar improvements from baseline in the control or alternative intervention group.

In an intervention delivering care for children with obesity and comparing face-to-face delivery with telehealth, parents rated telehealth consultations lower than face-to-face consultations when asked whether the provider explained things about the child’s health in a way that was easy to understand [43].

### Patient Outcomes

Of the 6 studies reporting patients’ clinical outcomes (Multimedia Appendix 4), 4 [10,28,33,34] found significant improvements (change compared with baseline, *P*<.05) after the intervention. Vismara et al [34] found that after speech therapy the rate of child vocalizations and their joint attention increased, while another study [33] found that child social communication behaviors improved following the use of an intervention comprising videoconferencing and learning modules. Among the 2 studies evaluating stand-alone digital communication using patients’ clinical outcomes [10,28], none showed a difference compared with the control group. In e-counselling for behavioral problems, Becker [28] found the frequency and severity of the child’s disruptive behaviors reduced at 2 weeks compared with baseline, but this difference was found in both the digital communication group and the telephone group. Similarly, in children with atopic dermatitis, the Objective Severity Scoring of Atopic Dermatitis scores were improved at 12 months in both the intervention (remote dermatology consultations via Web messaging) and control groups [10].

Digital communication was described as helpful by patients with traumatic brain injury [45] and as easy to use by the majority of patients using a portal for diabetes management [44].

### Health Professional Outcomes

In the 2 studies reporting health care professionals’ perceptions [20,30], their response was positive overall. In 1 study testing the feasibility of Skype and FaceTime updates with parents in the neonatal intensive care unit, 94% of providers rated the ease of using videoconferencing as excellent or good and more than 90% perceived videoconferencing to be reliable [30]. Similarly, in the home health care of premature infants, most nurses were motivated to use the information and communication technology; however, some were reluctant [20], feeling, for example, that the use of digital communication by the families should be discouraged in general, since these activities took families’ attention away from their infant.

### Health Service Delivery and Economic Outcomes

A total of 4 studies evaluated the effect of digital communication on health service delivery outcomes [10,37,38,46]. Of these, 2 reported the frequency of use, with 1 study of a Web-based tool for atopic dermatitis finding 8.3 messages sent per participant over 12 months [10], and 1 study of an SMS text messaging tool for caregivers of patients with disabilities finding 6.25 messages sent per participant over 3 months [46]. In the latter study, the content of digital communication was also described, with participants using SMS text messaging for social interaction and to ask questions [46]. Email was used predominantly to provide participants with information about common diseases and treatments. In an online portal for patients with chronic conditions, 64% of participants used the portal instead of calling their health care provider on at least one occasion [37,38].

In the Australian context, pediatric palliative care by videoconsultation at home saved costs versus face-to-face consultation during hospital-based consultations or during home visits [36].

There were 2 studies on parental management of children with atopic dermatitis evaluating economic outcomes [10,25]. One compared Web-based consultations versus a control group where participants were encouraged to seek treatment through traditional means [10]; except for hospital admissions, there were fewer health care visits (general practitioner visits, outpatient consultations, emergency visits, and complementary therapists visits) at 12 months in both groups, but there was no significant difference between the 2 groups. The other study reported that an eHealth portal was saving €594 per patient in the first year, mainly through a reduction in work absenteeism, the probability of eHealth reducing costs being 73% or greater after sensitivity analyses [25].

### Technical Problems

In 4 studies [29,30,34,44], participants reported experiencing technical problems. Vismara et al [34], who used telehealth for early autism training, found that all participants experienced some degree of frustration when using the videoconferencing program, including the audio or webcam not working or the Internet connection freezing. Passwords were reported as a barrier to using a diabetes Web portal and Web messaging [44], with the procedure for replacing lost passwords and creating one’s own password found to create problems. Using a videoconferencing system in the neonatal intensive care unit, some parents experienced technical problems, such as frozen screens, attributed to poor Internet connections at the parent’s home [30]. Finally, in a study focused on adolescent eating disorders [29], 3 out of 13 parents experienced technical problems, the nature of which was not described, during the Web chat sessions with a therapist.

## Discussion

### Principal Findings

The majority of studies were quantitative research, which explored the impact of digital communication in a broad range of conditions. Videoconsultations were often used. Most articles reported experiences of families of young children where the communication was with a parent. Findings of these studies of the use of digital clinical communication were equivocal, with no clear benefits in relation to patient, caregiver, or health care professional outcomes reported, but no adverse events reported either. Where digital clinical communication had been explored qualitatively, the key themes were the perception of the removal of barriers to communication and the formation of networks for communication facilitated by these technologies. Digital communications were found to be acceptable in most of the studies. Overall, parents were satisfied with their experience and perceived benefit.

Findings for economic benefit were equivocal: 2 studies found economic benefits of digital communication in comparison with face-to-face consultations, while 1 showed a neutral impact on resource use.

Our rapid review also had a specific focus on the use of digital communications by families and caregivers of young people in the transition period between pediatric and adult services. We had extended the age range for children and young people in this review to 26 years to include this period. Transition care is particularly challenging for the young person, the parent or caregiver, and the health care team [48]. Digital communication affords opportunities to young people to communicate privately with their health care team as they transition toward assuming greater responsibility for their health from their parents. Within the scope of the transition period, a greater emphasis could be placed on using digital technologies to enhance communication between health care professionals and patients directly, rather than through their parents. However, guidance indicates that the involvement of parents, combined with that of young people, is important at the transition stage [49,50]. This is why we were also interested in the use of digital communication between health professionals and parents during the transition period. Our rapid review showed that this population was absent from the identified literature, indicating a lack of evidence relating to the impact of family and caregiver involvement within this scope.

We are aware of a systematic review that assessed telehealth tools and interventions to support family caregivers of pediatric, adult, and older patients with chronic diseases [18]. This review of 64 articles published over 1997-2014 concluded that telehealth can positively affect care provided by family caregivers. Our work differs from the previous review in that we were interested only in the impact of digital clinical communication for families or caregivers of children or young people with health conditions; this excluded adults and older patients.

Other comprehensive reviews have investigated the impact of communication technologies on young people with mental health conditions [5] or with diabetes [9], and concluded that the benefit of using these technologies was unclear or inconclusive. However, these 2 reviews had no specific focus on family or caregiver involvement, which further justifies our work.

### Limitations and Strengths

Our rapid review presents several limitations. This work being a rapid review and not a systematic review [13], we chose not to undertake a gray literature search aimed at identifying unpublished studies, some of which may have failed to demonstrate the feasibility of these new technologies. Thus, the absence of publication bias in this area cannot be excluded. Overall, as we shortened time frames for literature searching and article retrieval, we may have missed some relevant information, which generates less certainty in our conclusions compared with using traditional systematic reviews [51]. As previously stated, we used here a rapid review method because the purpose of this type of review is to aid emergent decisions in health care settings.

As part of the rapid review process, we used simple checklists to assess study quality consistently and quickly. The use of detailed checklists may have enabled a more thorough quality assessment. However, as we were not excluding studies according to quality, we used these checklists in the context of a rapid review to give the reader an overview of the quality of the studies we included.

Many studies [19,21,31,38,44,47,52] examined interventions comprising more than one component, making it difficult to determine whether the results were due to the use of digital communication or other aspects of the intervention. For the purposes of this rapid review designed to scope the field using a narrative synthesis, we included these multifaceted studies, but future reviewers may wish to specifically limit their analysis to those studies where effects of the intervention can be separately assessed. In addition, we acknowledge that incorporating a wide range of outcomes does mean that some interpretation might be missed in narratively summarizing these, and focusing on particular outcome groupings of interest would allow for more in-depth synthesis.

As the included studies came from only 9 different countries, the generalizability of the findings beyond these settings is limited. Several of the studies came from the same groups of authors, working in specific clinical areas. These studies may therefore be indicative of clinical enthusiasts reporting their work. This has been observed in other reviews of digital interventions [3].

Although pre/post studies were not helpful to measure the direct impact of digital communication in the study populations, these studies were informative to address our objective to assess the feasibility of these technologies—that is, to verify their capacity to work correctly and be usable by health care professionals, families, and caregivers.

Regarding the assessment of feasibility, we emphasized in our quality assessment that a limitation of the studies was the uncertainty on whether the participants in RCTs and pre/post studies were representative of those in the general population, which again raises some issues of generalizability for these data. Indeed, the finding that some digital communication tools were usable by the included families and caregivers does not mean that the entire population would be able to use these with the same benefit. However, the use of digital communication technologies is becoming increasingly ubiquitous in our societies.

There is an emerging body of literature on the use of digital communication to improve families’ and caregivers’ involvement in the management of children and young people. Overall, these interventions show promise, but the evidence base to support them is lacking since, overall, we rated just 10 studies as good quality. Based on results from RCTs showing no differences between digital communication technologies and standard care, some authors suggest that such means of communication could be used to reach populations in underserved areas within large territories such the United States or Australia.

However, we would recommend further confirmatory studies using RCT designs to be conducted with a mid- to long-term perspective and using outcomes other than feasibility outcomes only. An RCT can be designed as mainly a noninferiority or superiority study. If the digital clinical communication is aimed at replacing an existing means of communication between health professional and families or caregivers, we believe a noninferiority design may be most appropriate. With such a design, the choice of the noninferiority margin, which corresponds to some loss of efficacy that might be accepted, could be easily justified by accounting for other benefits that digital clinical communication might have over standard care. If the use of digital clinical communication is added to standard care in one particular health condition in order to fulfill an unmet need, we believe a superiority design should be implemented. In this case, we would also recommend an economic evaluation to be conducted alongside so as to explore the economic impact the new intervention may have.

Given the absence of studies, future research could also be conducted with a specific focus on the use of digital communications in the transition period between pediatric and adult services given the special needs of this population [53,54].

### Conclusion

This rapid review showed an emerging body of literature suggesting the feasibility and acceptance of, and good rates of satisfaction with, digital technologies to enhance communication between health care professionals and the families or caregivers of children or young people under their care. However, we found no clear impact of these technologies on outcomes. Further evaluations based on comparative studies with larger sample sizes are needed to confirm these preliminary results and should investigate the impact of digital communication in terms of quality and organization of care, as well as the associated economic outcomes. An important topic for future research could be the evaluation of digital communication involving parents and caregivers in the management of young people during the transition from pediatric to adult health services.

## Appendix

Multimedia Appendix 1 Record of searches.

Multimedia Appendix 2 Characteristics of included studies.

Multimedia Appendix 3 Characteristics of included studies and quality assessment.

Multimedia Appendix 4 Summary of results.

Multimedia Appendix 5 Complete results.

## Abbreviations

pre/post studies: pre- and postintervention uncontrolled studies
PRISMA: Preferred Reporting Items for Systematic Reviews and Meta-Analyses
RCT: randomized controlled trial
SMS: short message service
