# Supplementary material for: Digital Clinical Communication for Families and Caregivers of Children or Young People With Short- or Long-Term Conditions: Rapid Review
Source: J Med Internet Res. 2018 Jan 5;20(1):e5. doi: 10.2196/jmir.7999 (PMC5775486; doi:10.2196/jmir.7999)
Supplement: Multimedia Appendix 1 [file jmir_v20i1e5_app1.pdf]

Multimedia Appendix 1: Record of searches

Record of main database searches for five of the six LYNC project rapid reviews

MEDLINE (Ovid) 1946 to August Week 3 2015, searched 27/08/2015

1	Electronic Mail/	2038
2	(email* or e-mail* or web-mail* or webmail* or internet-mail*).tw.	7666
3	Text Messaging/	887
4	(text messag* or texting or multimedia message*).tw.	1263
5	((mobile phone* or cellular phone* or cell phone*) and (message* or text* or sms or mms)).tw.	819
6	Social Media/	1923
7	(social media or social networking or blog* or facebook or myspace or twitter).tw.	3505
8	((internet* or web* or information or patient or health) adj2 (portal* or forum)).tw.	1673
9	(smartphone app* or smart phone app* or PDA app* or personal digital assistant app*).tw.	331
10	(video-conferenc* or videoconferenc* or videophone* or video-phone* or Voice over Internet Protocol or VoIP or skype or (google adj2 (talk or hangouts))).tw.	1810
11	Videoconferencing/	923
12	digital interactive television.tw.	5
13	1 or 2 or 3 or 4 or 5 or 6 or 7 or 8 or 9 or 10 or 11 or 12	17838
14	(digital or electronic or virtual or computer* or software* or internet* or online or on-line or web* or multimedia or multi-media or communication technolog* or telecommunication* or ICT or network* technolog* or telemedic* or	163183

	telecare or telehealth* or telepsychiatr*).ti.	
15	*computer communication networks/ or *internet/	37023
16	*telecommunications/ or *telemedicine/ or *remote consultation/ or *telepathology/ or *teleradiology/ or *cell phones/ or *modems/ or *wireless technology/	22303
17	13 or 14 or 15 or 16	200778
18	exp *Professional-Patient Relations/ or exp *Professional-Family Relations/	62797
19	((clinic* or center* or centre* or service* or hospital* or doctor* or physician* or clinician* or nurse* or pharmacist* or health worker* or professional* or provider* or practitioner* or therapist* or educator* or psychiatr* or patient* or outpatient* or out-patient* or inpatient* or in-patient* or client* or child* or teen* or paediatric* or pediatric* or boy* or girl* or youth* or schoolchild* or adoles* or minor or minors or under age* or juvenile* or schoolage* or school age* or young adult* or young person* or young people or student* or parent* or mother* or father* or brother* or sister* or sibling* or family or families or carer* or caregiver* or care giver*) adj4 (communicat* or relation* or interact* or convers* or discuss* or message* or feedback or respond* or response* or receive* or consult* or contact* or advice or advis* or counsel* or recommend* or monitor* or review* or diary or diaries or assess* or support* or educat* or train* or manage* or care or treat* or therapy or therapies or intervention* or report* or ongoing partnership)).tw.	2616393
20	18 or 19	2652071
21	17 and 20	41890
22	Young Adult/ or Adolescent/ or Child/ or Students/	2569792

23	(child* or teen* or paediatric* or pediatric* or boy* or girl* or youth* or schoolchild* or school child* or kid* or adoles* or minor or minors or under age* or juvenile* or pubescen* or secondary school* or highschool* or high school* or peer group* or schoolage* or school age* or young adult* or young person* or young people or student* or sixth form* or higher education or further education or undergraduate* or college* or universit*).tw.	2217902
24	22 or 23	3751056
25	21 and 24	15042

MEDLINE In-Process & Other Non-Indexed Citations (Ovid) September 02, 2015, searched 03/09/2015

1	Electronic Mail/	0
2	(email* or e-mail* or web-mail* or webmail* or internet-mail*).tw.	1509
3	Text Messaging/	0
4	(text messag* or texting or multimedia message*).tw.	457
5	((mobile phone* or cellular phone* or cell phone*) and (message* or text* or sms or mms)).tw.	257
6	Social Media/	0
7	(social media or social networking or blog* or facebook or myspace or twitter).tw.	1327
8	((internet* or web* or information or patient or health) adj2 (portal* or forum)).tw.	306
9	(smartphone app* or smart phone app* or PDA app* or personal digital assistant app*).tw.	222
10	(video-conferenc* or videoconferenc* or videophone* or video-phone* or Voice over Internet Protocol or VoIP or skype or (google adj2 (talk or hangouts))).tw.	240

11	Videoconferencing/	0
12	digital interactive television.tw.	1
13	2 or 4 or 5 or 7 or 8 or 9 or 10 or 12	3888
14	(digital or electronic or virtual or computer* or software* or internet* or online or on-line or web* or multimedia or multi-media or communication technolog* or telecommunication* or ICT or network* technolog* or telemedic* or telecare or telehealth* or telepsychiatr*).ti.	27501
15	13 or 14	30742
16	((clinic* or center* or centre* or service* or hospital* or doctor* or physician* or clinician* or nurse* or pharmacist* or health worker* or professional* or provider* or practitioner* or therapist* or educator* or psychiatr* or patient* or outpatient* or out-patient* or inpatient* or in-patient* or client* or child* or teen* or paediatric* or pediatric* or boy* or girl* or youth* or schoolchild* or adoles* or minor or minors or under age* or juvenile* or schoolage* or school age* or young adult* or young person* or young people or student* or parent* or mother* or father* or brother* or sister* or sibling* or family or families or carer* or caregiver* or care giver*) adj4 (communicat* or relation* or interact* or convers* or discuss* or message* or feedback or respond* or response* or receive* or consult* or contact* or advice or advis* or counsel* or recommend* or monitor* or review* or diary or diaries or assess* or support* or educat* or train* or manage* or care or treat* or therapy or therapies or intervention* or report* or ongoing partnership)).tw.	252369
17	15 and 16	5700
18	(child* or teen* or paediatric* or pediatric* or boy* or girl* or youth* or schoolchild* or school child* or kid* or adoles* or minor or minors or under age* or juvenile* or pubescen* or secondary school* or highschool* or high school* or peer group* or schoolage* or school age* or young adult* or young person* or young people or student* or sixth form* or higher education or further education or undergraduate* or college* or universit*).tw.	191893
19	17 and 18	1938

Embase (Ovid) 1974 to 2015 Week 35, searched 02/09/2015

1	*e-mail/	1150
2	(email* or e-mail* or web-mail* or webmail* or internet-mail*).tw.	18033
3	*text messaging/	780
4	(text messag* or texting or multimedia message*).tw.	2242
5	((mobile phone* or cellular phone* or cell phone*) and (message* or text* or sms or mms)).tw.	1469
6	*social media/	2002
7	(social media or social networking or blog* or facebook or myspace or twitter).tw.	6596
8	((internet* or web* or information or patient or health) adj2 (portal* or forum)).tw.	2702
9	(smartphone app* or smart phone app* or PDA app* or personal digital assistant app*).tw.	866
10	(video-conferenc* or videoconferenc* or videophone* or video-phone* or Voice over Internet Protocol or VoIP or skype or (google adj2 (talk or hangouts))).tw.	2742
11	*videoconferencing/	576
12	digital interactive television.tw.	7
13	1 or 2 or 3 or 4 or 5 or 6 or 7 or 8 or 9 or 10 or 11 or 12	33230
14	(digital or electronic or virtual or computer* or software* or internet* or online or on-line or web* or multimedia or multi-media or communication technolog* or telecommunication* or ICT or network* technolog* or telemedic* or	219504

	telecare or telehealth* or telepsychiatr*).ti.	
15	*computer network/ or *internet/	34588
16	exp *telehealth/ or *teleconsultation/ or *mobile phone/	18931
17	13 or 14 or 15 or 16	267290
18	*doctor patient relation/ or *nurse patient relationship/	47263
19	((clinic* or center* or centre* or service* or hospital* or doctor* or physician* or clinician* or nurse* or pharmacist* or health worker* or professional* or provider* or practitioner* or therapist* or educator* or psychiatr* or patient* or outpatient* or out-patient* or inpatient* or in-patient* or client* or child* or teen* or paediatric* or pediatric* or boy* or girl* or youth* or schoolchild* or adoles* or minor or minors or under age* or juvenile* or schoolage* or school age* or young adult* or young person* or young people or student* or parent* or mother* or father* or brother* or sister* or sibling* or family or families or carer* or caregiver* or care giver*) adj4 (communicat* or relation* or interact* or convers* or discuss* or message* or feedback or respond* or response* or receive* or consult* or contact* or advice or advis* or counsel* or recommend* or monitor* or review* or diary or diaries or assess* or support* or educat* or train* or manage* or care or treat* or therapy or therapies or intervention* or report* or ongoing partnership)).tw.	3905370
20	18 or 19	3933505
21	17 and 20	63407
22	exp student/	91612
23	(child* or teen* or paediatric* or pediatric* or boy* or girl* or youth* or schoolchild* or school child* or kid* or adoles* or minor or minors or under age* or juvenile* or pubescen* or secondary school* or highschool* or high school* or peer group* or schoolage* or school age* or young adult* or young person* or young people or student* or sixth form* or higher education or further education or undergraduate* or college* or universit*).tw.	3188530

24	limit 21 to (child <unspecified age> or school child <7 to 12 years> or adolescent <13 to 17 years>)	8020
25	22 or 23	3205416
26	21 and 25	20498
27	24 or 26	22140

Cochrane Library (Wiley), searched 02/09/2015

#1	MeSH descriptor: [Electronic Mail] this term only	208
#2	(email* or e-mail* or webmail* or web-mail* or internet-mail*):ti,ab,kw	1042
#3	MeSH descriptor: [Text Messaging] this term only	161
#4	(text next messag* or texting or multimedia next message*):ti,ab,kw	529
#5	((mobile next phone* or cellular next phone* or cell next phone*) and (message* or text* or sms or mms)):ti,ab,kw	334
#6	MeSH descriptor: [Social Media] this term only	26
#7	("social media" or "social networking" or blog* or facebook or myspace or twitter):ti,ab,kw	190
#8	((internet* or web* or information or patient or health) near/2 (portal* or forum)):ti,ab,kw	110
#9	(smartphone next app* or "smart phone" next app* or PDA next app* or "personal digital assistant" next app*):ti,ab,kw	69
#10	(video-conferenc* or videoconferenc* or videophone* or video-phone* or "Voice over Internet Protocol" or VoIP or skype or (google near/2 (talk or hangouts))):ti,ab,kw	318
#11	MeSH descriptor: [Videoconferencing] this term only	98
#12	"digital interactive television":ti,ab,kw	1
#13	#1 or #2 or #3 or #4 or #5 or #6 or #7 or #8 or #9 or #10 or #11 or #12	2260
#14	(digital or electronic or virtual or computer* or software* or internet* or online or on-line or web* or multimedia or multi-media or communication next technolog* or telecommunication* or ICT or network* next technolog* or telemedic* or telecare or telehealth* or telepsychiatr*):ti	10980
#15	[mh ^"computer communication networks" [mj]] or [mh ^internet [mj]]	1045
#16	[mh ^telecommunications [mj]] or [mh ^telemedicine [mj]] or [mh ^"remote consultation" [mj]] or [mh ^telepathology [mj]] or [mh ^teleradiology [mj]] or [mh ^"cell phones" [mj]] or [mh ^modems [mj]] or [mh ^"wireless technology" [mj]]	528
#17	#13 or #14 or #15 or #16	12873
#18	[mh "Professional-Patient Relations" [mj]] or [mh "Professional-Family	1808

	Relations" [mj]]	
#19	((clinic* or center* or centre* or service* or hospital* or doctor* or physician* or clinician* or nurse* or pharmacist* or health next worker* or professional* or provider* or practitioner* or therapist* or educator* or psychiatr* or patient* or outpatient* or out-patient* or inpatient* or in-patient* or client* or child* or teen* or paediatric* or pediatric* or boy* or girl* or youth* or schoolchild* or adoles* or minor or minors or under next age* or juvenile* or schoolage* or school next age* or young next adult* or young next person* or young next people or student* or parent* or mother* or father* or brother* or sister* or sibling* or family or families or carer* or caregiver* or care next giver*) near/4 (communicat* or relation* or interact* or convers* or discuss* or message* or feedback or respond* or response* or receive* or consult* or contact* or advice or advis* or counsel* or recommend* or monitor* or review* or diary or diaries or assess* or support* or educat* or train* or manage* or care or treat* or therapy or therapies or intervention* or report* or ongoing next partnership)):ti,ab,kw	347291
#20	#18 or #19	347294
#21	#17 and #20	6476
#22	[mh ^"Young Adult"] or [mh ^Adolescent] or [mh ^Child] or [mh ^Students]	78701
#23	(child* or teen* or paediatric* or pediatric* or boy* or girl* or youth* or schoolchild* or school next child* or kid* or adoles* or minor or minors or under next age* or juvenile* or pubescen* or secondary next school* or highschool* or high next school* or peer next group* or schoolage* or school next age* or young next adult* or young next person* or young next people or student* or sixth next form* or higher next education or further next education or undergraduate* or college* or universit*):ti,ab,kw	231913
#24	#22 or #23	231913
#25	#21 and #24	2781

All Results (2781)

Cochrane Reviews (39)

Other Reviews (61)

Trials (2588)

Methods Studies (67)

Technology Assessments (6)

Economic Evaluations (20)

Cochrane Groups (0)

PsycINFO (Proquest), searched 08/09/2015

n.b. Results exported from line 24. Line S24 Limited to 2009 onwards due to Proquest restrictions on the number of records that can be exported. Unable to export results from line S21.

S24	S17 AND S22<br><br>Limited by:<br><br>Publication date after 2009	2,957*
S23	S17 AND S22	4,707*
S22	S18Limits applied	188,824*
S21	S19 AND S20	9,421*
S20	TI,AB(child* OR teen* OR paediatric* OR pediatric* OR boy* OR girl* OR youth* OR schoolchild* OR school PRE/0 child* OR kid* OR adoles* OR minor OR minors OR under PRE/0 age* OR juvenile* OR pubescen* OR secondary PRE/0 school* OR highschool* OR high PRE/0 school* OR peer PRE/0 group* OR schoolage* OR school PRE/0 age* OR young PRE/0 adult* OR young PRE/0 person* OR young PRE/0 people OR student* OR sixth PRE/0 form* OR higher PRE/0 education OR further PRE/0 education OR undergraduate* OR college* OR universit*)	1,245,671*
S19	S17 AND S18	15,817*
S18	TI,AB((clinic* OR center* OR centre* OR service* OR hospital* OR doctor* OR physician* OR clinician* OR nurse* OR pharmacist* OR health PRE/0 worker* OR professional* OR provider* OR practitioner* OR therapist* OR educator* OR psychiatr* OR patient* OR outpatient* OR out-patient* OR inpatient* OR in-patient* OR client* OR child* OR teen* OR paediatric* OR pediatric* OR boy* OR girl* OR youth* OR schoolchild* OR adoles* OR minor OR minors OR under PRE/0 age* OR juvenile* OR schoolage* OR school PRE/0 age* OR young PRE/0	642,259*

	adult* OR young PRE/0 person* OR young PRE/0 people OR student* OR parent* OR mother* OR father* OR brother* OR sister* OR sibling* OR family OR families OR carer* OR caregiver* OR care PRE/0 giver*) PRE/3 (communicat* OR relation* OR interact* OR convers* OR discuss* OR message* OR feedback OR respond* OR response* OR receive* OR consult* OR contact* OR advice OR advis* OR counsel* OR recommend* OR monitor* OR review* OR diary OR diaries OR assess* OR support* OR educat* OR train* OR manage* OR care OR treat* OR therapy OR therapies OR intervention* OR report* OR ongoing PRE/0 partnership))	
S17	S10 OR S11 OR S12 OR S13 OR S14 OR S15 OR S16	84,172*
S16	MJSUB.EXACT("Online Therapy")	1,492°
S15	MJSUB.EXACT("Telemedicine")	2,614°
S14	MJSUB.EXACT("Internet")	19,034*
S13	MJSUB.EXACT("Cellular Phones")	2,003°
S12	MJSUB.EXACT("Websites")	2,602°
S11	TI(digital OR electronic OR virtual OR computer* OR software* OR internet* OR online OR on-line OR web* OR multimedia OR multi-media OR communication PRE/0 technolog* OR telecommunication* OR ICT OR network* PRE/0 technolog* OR telemedic* OR telecare OR telehealth* OR telepsychiatr*)	63,637*
S10	S1 OR S2 OR S3 OR S4 OR S5 OR S6 OR S7 OR S8 OR S9	22,147*
S9	MJSUB.EXACT.EXPLODE("Electronic Communication")	10,148*
S8	TI,AB("digital interactive television")	4°
S7	TI,AB(video-conferenc* OR videoconferenc* OR videophone* OR video-phone* OR "Voice over Internet Protocol" OR VoIP OR skype OR (google PRE/1 (talk OR hangouts)))	1,466°
S6	TI,AB((smartphone PRE/1 app* OR “smart phone” PRE/1 app* OR PDA PRE/1 app* OR “personal digital assistant” PRE/1 app*))	169°
S5	TI,AB((internet* OR web* OR information OR patient OR health) NEAR/1 (portal* OR forum))	745°
S4	TI,AB("social media" OR "social networking" OR blog* OR facebook OR myspace OR twitter)	7,850*
S3	TI,AB((mobile PRE/0 phone* OR cellular PRE/0 phone* OR cell PRE/0 phone*) AND (message* OR text* OR sms OR mms))	766°
S2	TI,AB(text PRE/0 messag* OR texting OR multimedia PRE/0 message*)	1,342°
S1	TI,AB(email* OR e-mail* OR web-mail* OR webmail* OR internet-mail*)	6,408*

Record of searching and sorting within EndNote for this rapid review

Families / Carers

In the EndNote Library of results from the above searches, we clicked on ‘Rapid reviews main search > All’ and in the search panel we selected ‘Search Whole Group’

	Title field	Contains	parent*
Or	Title field	Contains	mother*
Or	Title field	Contains	father*
Or	Title field	Contains	sibling*
Or	Title field	Contains	brother*
Or	Title field	Contains	sister*
Or	Title field	Contains	family
Or	Title field	Contains	families
Or	Title field	Contains	carer*
Or	Title field	Contains	caregiver*
Or	Title field	Contains	care-giver*

Records from database searching (2009 onwards): 1146

Records from other sources: 10

Total: 1156
