# Supplementary material for: Digital Clinical Communication for Families and Caregivers of Children or Young People With Short- or Long-Term Conditions: Rapid Review
Source: J Med Internet Res. 2018 Jan 5;20(1):e5. doi: 10.2196/jmir.7999 (PMC5775486; doi:10.2196/jmir.7999)
Supplement: Multimedia Appendix 2 [file jmir_v20i1e5_app2.pdf]

Multimedia Appendix 2. Characteristics of included studies.

Study design, Author, Year	Country	Participants	Intervention using the digital clinical communication	Control group	Quality rating
Randomized controlled trials (RCTs) : n=10					
Bergmo et al. [10], 2009	Norway	Parents of 98 children with atopic dermatitis	Remote dermatology consultations	Usual care	Good
Grover et al. [19], 2011	UK	64 carers of people with Anorexia Nervosa	Carers participating in a web- based therapy sessions followed by email or phone support from a clinician	Usual care	Poor
Gund et al. [20], 2013	Sweden	34 families of preterm babies	A web application allowing families to communicate via a web messaging service- video conferences with nurses	Standard home health care (phone calls)	Fair
Hanberger et al. [21], 2013	Sweden	474 families of children with type 1 diabetes	A web- based portal allowing communication	Usual care (no access to the web-based portal)	Good
Looman et al. [22], 2015	USA	163 families of children with medical complexity receiving paediatric health care home	Access to an advanced practice registered nurse (APRN) care co-ordinator by telephone and video conferencing	Access to APRN by telephone only / Another control group who could contact the clinics general telephone number	Good
Petranovich et al. [23], 2015	USA	132 families of adolescents with traumatic brain injury (TBI)	A counsellor-assisted problem solving intervention (CAPS) where families completed online modules and participated in skype sessions	An internet resource comparison program (IRC)	Fair
Scharer et al. [24], 2009	USA	11 mothers/caregivers of children with serious mental illness	Web-based social support with a chat room	Telephone social support	Good
Van Os-Medendorp et al. [25], 2012	Netherlands	90 parents of children with atopic dermatitis	A web-portal which allowed e-consultations with a nurse	Face-to-face care	Good

Wade et al. [26], 2014	USA	132 families of adolescents with TBI	A CAPS where families participated in online modules and skype sessions	An IRC program	Fair
Wade et al. [27], 2012	USA	41 families of adolescents with TBI	The Teen Online Problem Solving (TOPS) intervention (self-guided online modules and video conferencing)	An IRC program	Fair
Controlled trial (non-randomised): n=2					
Becker et al. [28], 2012	USA	63 parents/carers of healthy children	A parent advice line offered two nights per week with possibility to call or email the service	Phone call	Fair
Bradford et al.[11], 2012	Australia	14 parents of children referred to a Paediatric Palliative Care Service (PPCS)	Telehealth consultation in addition to usual care	Usual care (face to face care and PPCS nursing care services)	Fair
Pre/post uncontrolled study: n=7					
Binford Hopf et al. [29], 2013	USA	13 parents of 10 children with eating disorders	Group web-chat sessions with fellow parents and a clinical psychologist	/	Fair
Epstein et al. [30], 2015	USA	26 parents of patients in the NICU	Parents receiving daily Skype or FaceTime updates from staff	/	Good
Gulmans et al. [31], 2012	Netherlands	30 parents of children with cerebral palsy	A web based system for parent- professional communication and inter- professional communication	/	Fair
Haney et al. [32], 2012	USA	19 parents and carers of children various medical conditions	Emails for parents/ caregivers to communicate with nurses	/	Fair
Vismara et al. [33], 2013	USA	Parents of 8 children with Autism spectrum disorder (ASD)	Video conferencing with a therapist and a website allowing parents to access text and video based learning modules and message boards	/	Fair
Vismara et al. [34], 2012	USA	Parents of 9 children with ASD	Video conferencing with a therapist and a parent intervention curriculum delivered by telehealth	/	Fair

Wade et al. [35], 2009	USA	Families of 9 children with TBI	Video- conferencing and a website containing links to resources and self-guided session materials	/	Fair
Economic model: n=1					
Bradford et al. [36], 2014	Australia	95 home video paediatric palliative care consultations	Home video consultations (home telehealth program)	Face to face consultatio ns	Good
Cross-sectional & observational: n=9					
Braverman et al [37], 2011	Russia	70 parents of children with kidney diseases	An educational website allowing parents to ask questions, upload documents and provide information	/	Fair
Byczkowski et al. [38], 2014	USA	126 parents of children with various long term conditions	Condition specific patient portals providing access to medical record elements and allowing secure messaging	/	Fair
de Graaf et al. [39], 2013	Netherlands	128 carers of children with Infantile haemangioma	eHealth intervention including e-learning and e-consults with a dermatologist	/	Fair
Hanlon-Dearman et al. [40], 2014	Canada	16 families of children diagnosed with foetal alcohol spectrum disorder	Telehealth (diagnostic assessment and/or individual or group follow up)	/	Good
Hopper et al. [41], 2011	Australia	10 carers of children referred to a genetics service	Video recorded consultations with a genetic counsellor with a live-feed of the session delivered to the clinical geneticist	/	Poor
Lee et al. [42], 2010	USA	42 parents of infants with retinopathy of prematurity	Wide-field retinal imaging sent to a remote expert via telemedicine	/	Fair
Mulgrew et al. [43], 2011	USA	Parents of children with obesity	Rural/remote clinic received telemedicine consultations with a paediatrician	Face to face consultatio ns	Fair
Nordfeldt et al. [44], 2010	Sweden	19 parents of children with diabetes and 5 children with diabetes	Portal providing diabetes information, blogs and message boards	/	Good
Wade et al. [45], 2009	USA	9 families of children with TBI	The TOPS intervention (self-guided online modules and video conferencing)	/	Fair

Interview studies: n=2					
Barlott et al. [46], 2015	Columbia	8 parents of people with disabilities	SMS to receive information, ask questions and for social interaction	/	Fair
Britto et al. [47], 2013	USA	24 parents of 25 children with various long term conditions	Condition specific patient portals providing access to medical record elements and allowing secure messaging	/	Good
