# Supplementary material for: Digital Clinical Communication for Families and Caregivers of Children or Young People With Short- or Long-Term Conditions: Rapid Review
Source: J Med Internet Res. 2018 Jan 5;20(1):e5. doi: 10.2196/jmir.7999 (PMC5775486; doi:10.2196/jmir.7999)
Supplement: Multimedia Appendix 3 [file jmir_v20i1e5_app3.pdf]

**Multimedia Appendix 3.** Characteristics of included studies and quality assessment.

| Author, Year, Country, Study design | Participants | Intervention using the digital clinical communication technology | Comparison | Quality assessment – Main limitation |
|---|---|---|---|---|
| Barlott et al. [46], 2015, Columbia, Interview study | 8 parents of people with disabilities. Children aged 7-32 | SMS messaging used for parents to receive information, ask questions and for social interaction | None | **Fair -** Unsure if relationship between researcher and participants was adequately considered |
| Becker et al. [28], 2012, USA, Non-randomised controlled study | 63 parent/carers of healthy children. Child mean age 6.5 years. Phone call group (n= 33) Email group (n=30) | A parent advice line, staffed by an on call licensed psychologist and two doctoral students, offered two nights per week. Possibility for parents to call or email the service about common developmental problems (e.g. bed wetting, tantrums) | Parents who contacted the service by phone | **Fair –** Lost-to-follow-up > 20% |
| Bergmo et al. [10], Norway, RCT | Parents of 98 children with atopic dermatitis. Mean child age in intervention group = 4.6 and in control group = 5.3. Intervention (n=50) Control (n=48) | Remote dermatology consultations where parents could send photographs of their child's eczema with a written description of the condition, and the specialist could respond with treatment advice | A control group receiving usual care | **Good -** No blinded assessment of outcomes |
| Binford Hopf et al. [29], 2013, USA, Pre/post intervention uncontrolled | 13 parents of 10 children with eating disorders aged 9-17 | Parents' participation in 15 group web-chat sessions with fellow parents and a clinical psychologist | Same group compared before and after the intervention | **Fair** - Small sample size |
| Bradford et al. [36], 2014, Australia, Economic model | 95 home video paediatric palliative care consultations | Home video consultations (home telehealth program) for which actual costs were calculated. This was compared to the estimated costs of the consultations had they been conducted face to face | Estimated costs of face to face consultations | **Good-** Costs not directly translatable to UK but may be similar |
| Bradford et al. [11], 2012, Australia, Non-randomised | 14 parents of children (aged 0- 18) referred to a Paediatric Palliative Care | Telehealth consultation (symptom management, discussions of changes in the patient's and | Usual care (face to face care and the organisation of community | **Fair -** Unsure if study participants were representative of those eligible in |

| | | | | |
|---|---|---|---|---|
| controlled study | Service (PPCS).<br>Intervention (n=6)<br>Control (n=6)<br>Not allocated (n=2) | emotional support for caregivers) in addition to usual care | nursing care services by PPCS staff) | the general population |
| Braverman et al [37], 2011, Russia, Cross-sectional study | 70 parents of children (aged 1 month – 17 years) with patients with kidney diseases | An educational website for parents of children with kidney diseases allowing parents to ask questions, upload documents (e.g. medical summaries) and provide information about the child's diagnosis | A Paediatric Nephrologist compared the information they received to the child's diagnosis | **Fair-** Sample size justification, power and effect sizes not reported |
| Britto et al. [47], 2013, USA, Interview study | 24 parents of 25 children with long term conditions (cystic fibrosis, diabetes mellitus, juvenile idiopathic arthritis) | Condition specific patient portals providing access to medical record elements (e.g. test results) and allowing secure messaging with care providers | None | **Good-** Relationship between researcher and participants not adequately considered |
| Byczkowski et al. [38], 2014, USA, Cross-sectional Study | 126 parents of children with long term conditions (cystic fibrosis, diabetes mellitus, juvenile idiopathic arthritis) who used the web based portal and 15 parents who obtained an account for the portal but used it less than 3 times | Condition specific patient portals providing access to medical record elements (e.g. test results) and allowing secure messaging with care providers | None | **Fair -** Sample size justification, power and effect sizes not reported |
| de Graaf et al. [39], 2013, Netherlands, Cross-sectional Study | 128 carers (127 parents, 1 grandparent) of children with Infantile haemangioma | eHealth intervention including e-learning and e-consults (parents submitting a photograph of their child's skin lesion and information about its growth pattern – advice provided by a dermatologist on diagnosis and risk of complications and need to see a specialist) | None | **Fair -** Sample size justification, power and effect sizes not reported |
| Epstein et al. [30], 2015, USA, Pre/post intervention uncontrolled with interview study | 26 parents of patients in the NICU | Parents receiving daily Skype or facetime updates from staff in the NICU once a day for five days, the content of updates being similar to those usually provided by phone (feedings events of the day, parents questions answered) | Same group compared before and after the intervention | **Good -** Unsure if study participants were representative of those eligible in the general population, Outcome measures of interest not taken multiple times before the intervention and multiple times after |

| | | | | |
|---|---|---|---|---|
| | | | | the intervention |
| Grover et al. [19], 2011, UK, RCT | 64 carers of people with Anorexia Nervosa aged 12- 44. Intervention (n=34) Control (n=30) | Carers participating in a web- based therapy sessions followed by email or phone support from a clinician | Usual care (support from the organisation Beat) | **Poor-** Estimate of the treatment effect was not precise |
| Gulmans et al. [31], 2012, Netherlands, Pre/post intervention uncontrolled | 30 parents of children with cerebral palsy aged between 4 and 8 years | A web based system for parent- professional communication and inter- professional communication, where parents could ask questions and review their communication from professionals | Same group compared before and after the intervention | **Fair -** Unsure if study participants were representative of those who would be eligible in the general population |
| Gund et al. [20], 2013, Sweden, RCT | 34 families of preterm babies. Median infant gestational age = 33 weeks + 5 days. Web application (n=12) Video - conferencing (n=9) Control (n=13) | A web application allowing families to communicate with healthcare professionals via a web messaging service- families had video conferences with nurses instead of phone calls (standard home health care) | Another group with standard hone health care and a control group receiving standard home health care after discharge | **Fair -** No measure of the intervention effect |
| Hanberger et al. [21], 2013, Sweden, RCT | 474 families of children with type 1 diabetes. Mean child age in the intervention group =13.2. Mean child age in the control group =13.3. Intervention (n=244) Control (n=230) | A web- based portal which provided diabetes related information and allowed communication with diabetes peers and healthcare professionals. All parents in intervention group had access to the portal as did children over the age of 13 | A control group who had no access to the web-based portal for the first year of the study | **Good -** Unsure if results can be applied in another context/local population |
| Haney et al. [32], 2012, USA, Pre/post intervention uncontrolled | 19 parents and carers of children various medical conditions (severe birth asphyxia, TBI, severe cerebral palsy, trisomy 18, …). Mean child age 9.17 | Emails for parents/ caregivers to communicate with nurses who sent parent/carer givers a topic at the start of the week related to caring for child at home. Parents could sent questions, comments and concerns | Same group compared before and after the intervention | **Fair -** Unsure if study participants were representative of those who would be eligible in the general population |
| Hanlon-Dearman et al. [40], 2014, Canada, Cross-sectional Study | 16 families of children diagnosed with foetal alcohol spectrum disorder | Telehealth where families participated in diagnostic assessment and/or individual or group follow up | None | **Good-** Unsure if relationship between researcher and participants was adequately |

| | | | | |
|---|---|---|---|---|
| | | | | considered |
| Hopper et al. [41], 2011, Australia, Observational study (Survey of carers and consultation content) | 10 carers of children referred to a genetics service (children aged between 8 and 14) | Video recorded consultations with a genetic counsellor with a live-feed of the session delivered to the clinical geneticist. DVDs of the session and still photos were also sent to the geneticist | Face to face consultations conducted with the same patients, carers, genetics counsellor and clinical geneticist | **Poor -** Results are provided with very few details |
| Lee et al. [42], 2010, USA, Cross-sectional Study | 42 parents of infants who underwent wide-field retinal imaging for retinopathy of prematurity | Data from wide-field retinal imaging for retinopathy of prematurity being sent to a remote expert via telemedicine | None | **Fair -** Sample size justification, power and effect size not reported |
| Looman et al. [22], 2015, USA, RCT | 163 families of children with medical complexity receiving care form a special needs clinic (paediatric health care home).<br>Children aged between 2 and 15 years at randomisation.<br>Intervention (n=54)<br>Intervention + video conferencing (n=54)<br>Control (n= 55) | Access to an advanced practice registered nurse (APRN) care co-ordinator by telephone and video conferencing in the other telephone in one group | A group with an access to APRN by telephone only and a control group who could contact the clinics general telephone number for care coordination | **Good -** No statement related to the randomisation procedure |
| Mulgrew et al. [43], 2011, USA, Cross-sectional Study | Parents of children who had received consultations for childhood obesity. Patients were aged between 4 and 11 years.<br>Telemedicine (n=10)<br>Face to face (n=15) | Participants attending a rural/remote clinic received telemedicine consultations with a paediatrician specialising in weight management and a dietician. A rural healthcare provider was present with the patient and family during the consultation | Parent receiving face to face consultations | **Fair -** Sample size justification, power and effect size provided |
| Nordfeldt et al. [44], 2010, Sweden, Cross-sectional Study | 19 parents of children with diabetes and 5 children with diabetes aged between 11 and 18 | Launch of a portal for patients and parents of patients with type 1 diabetes that provided diabetes information, blogs and message boards | None | **Good -** none |

| | | | | |
|---|---|---|---|---|
| Petranovich et al. [23], 2015, USA, RCT | 132 families of adolescents with traumatic brain injury At baseline mean patient age was 14.7 in the CAPS group and 14.99 in the IRC group<br>CAPS (n=65)<br>IRC (n=67) | A counsellor-assisted problem solving intervention (CAPS) where families completed online modules and participated in skype sessions with the therapist | An internet resource comparison program (IRC) | **Fair –** Lost-to-follow-up > 20% |
| Scharer et al. [24], 2009, USA, RCT | 11 mothers (and maternal caregivers) of children with serious mental illness. Mean child age 9.82.<br>Web based support (n=7)<br>Telephone support (n=4) | Web-based social support with a chat room once a week for one hour (Chats involving one mother and the nurse were analysed) that was facilitated by a psychiatric nurse<br>In one group, patients received telephone social support | Telephone social support on a one to one basis from a psychiatric nurse every 2 weeks | **Good -** Unsure if ethical issues been taken in to consideration |
| Van Os-Medendorp et al. [25], 2012, Netherlands, RCT | 90 parents of children with atopic dermatitis. Mean child age in: intervention group = 2.9 control group = 2.7<br>Intervention (n=45)<br>Control (n=45) | An eczema web-portal which allowed e-consultations with a dermatology nurse and provided internet-guided monitoring and self-management training | Same group compared before and after the intervention | **Good -** Limited time horizon (1 year) |
| Vismara et al. [33], 2013, USA, Pre/post intervention uncontrolled | Parents of 8 children with Autism spectrum disorder. Mean child age – 27.5 months | Video conferencing with a therapist and a website allowing parents to access text and video based learning modules and message boards | Same group compared before and after the intervention | **Fair -** Very small sample size (quasi-experimental study) |
| Vismara et al. [34], 2012, USA, Pre/post intervention uncontrolled | Parents of 9 children with Autism spectrum disorder. Mean child age – 28.89 months | Video conferencing with a therapist and a parent intervention curriculum delivered by telehealth | Same group compared before and after the intervention | **Fair -** Very small sample size (quasi-experimental study) |
| Wade et al. [26], 2014, USA, RCT | 132 families of adolescents with traumatic brain injury. At the time of injury mean patient age was 14.7 in the IRC group and 14.40 in the CAPS group<br>CAPS (n=65).<br>IRC (n=67) | A counsellor-assisted problem solving intervention (CAPS) where families participated in online modules and skype sessions with the therapist | An internet resource comparison program (IRC) – a home page with links to online resources | **Fair-** Sample size justification, and power not reported |
| Wade et al. [35], 2009, USA, Pre/post intervention uncontrolled | Families of 9 children aged between 3 and 8 with traumatic brain injury | Video- conferencing and a website containing links to resources and self-guided session materials | Same group compared before and after the intervention | **Fair-** Unsure if study participants were representative of those eligible in the general population |

| | | | | |
|---|---|---|---|---|
| Wade et al. [27], 2012, USA, RCT | 41 families of adolescents aged 11 -18 with traumatic brain injury. TOPS (n=20) IRC (n=21) | The Teen Online Problem Solving (TOPS) intervention that included self-guided online modules and video conferencing with a therapist | An internet resource comparison program (IRC) – a home page with links to online resources | **Fair -** No information related to the method of randomisation |
| Wade et al. [45], 2009, USA, Cross – sectional study | 9 families of children with traumatic brain injury. Mean child age 15.04 years | The Teen Online Problem Solving (TOPS) intervention included self-guided online modules and video conferencing with a therapist | None | **Fair-** Unsure if study participants were representative of those eligible in the general population |
