# Supplementary material for: Digital Clinical Communication for Families and Caregivers of Children or Young People With Short- or Long-Term Conditions: Rapid Review
Source: J Med Internet Res. 2018 Jan 5;20(1):e5. doi: 10.2196/jmir.7999 (PMC5775486; doi:10.2196/jmir.7999)
Supplement: Multimedia Appendix 4 [file jmir_v20i1e5_app4.pdf]

Multimedia Appendix 4. Summary of results[a]

Study type, Author, Year	Family/carer outcomes	Patient outcomes	Health service outcomes
Randomized controlled trials (RCTs)			
Bergmo et al. [10], 2009	No differences between groups		
Grover et al. [19], 2011	78.8% of carers requested email support as part of the package and 21.2% preferred telephone support. No other parent/carer outcome specifically related to the digital communication tool	No outcomes specifically related to the digital communication tool	
Gund et al. [20], 2013	A majority of positive opinion (75% to 100%) regarding the use of the web-based system or skype calls with to some extent the possibility to reduce the need of home visits (33% with Web-based system and 75% with Skype)	None	
Hanberger et al. [21], 2013	No differences between groups		None
Looman et al. [22], 2015	None		Personal doctor rating and adequacy of care coordination rated higher in both intervention groups (telephone and video) as compared to control group (P=.001 and P=.026, respectively)
Petranovich et al. [23], 2015	No outcomes related to the communication component of the program		
Scharer et al. [24], 2009	Mothers in the chat room intervention group had to be active in seeking support (e.g. by signing into the chat room to access the nurse). In the telephone intervention group both active and passive mothers received consistent support. No quantitative outcomes	None	
Van Os-Medendorp et al. [25], 2012	None	No differences between groups on the severity of atopic dermatitis and Quality of Life	Total costs (direct + indirect) at 12 months were €3378 for patients using individualised e-health and €3972 for patients

			with usual care (face-to-face care), cost difference=-€594 (95%CI -€2545 to €1227)
Wade et al. [26], 2014	Participants who completed 5 or more CAPS sessions reported a greater reduction in depression that participants in the IRC intervention group (P =.03)	None	
Wade et al. [27], 2012	No results provided regarding the helpfulness of skype videoconferences	None	
Controlled trial (non-randomised)			
Becker et al. [28], 2012	No differences between groups		None
Bradford et al.[11], 2012	No differences between groups	None	
Pre/post uncontrolled study			
Binford Hopf et al. [29], 2013,	Improvement on the Eating Disorder Symptom Impact Scale total score (p=0.02), on the stigma (P=.02), and dependency (P=.05) subscales of the experience of Caregiving Inventory. No differences on the difficult behaviours (P=.30), negative symptoms (P=.44), problems with services (P=.33), effects on family (P=.06), need to backup (P=.59), loss (P=0.55), positive personal experience (P=.58) or good aspects of relationship (P=.48) subscales	None	
Epstein et al. [30], 2015	No difference on parents' understanding survey scores / baseline	None	
Gulmans et al. [31], 2012	70% of parents responded that the system considerably contributed to sufficient contact and 65% responded it considerably contributed to timely information exchange; 65% considered that the system considerably contributed to better accessibility for consultation	None	

Haney et al. [32], 2012	No improvement of the Pediatric Quality of Life subscales family impact and healthcare satisfaction / baseline	None	
Vismara et al. [33], 2013	Increase of the Maternal Behaviour Rating Scale score / baseline	Increase of vocalizations, joint attention initiations, vocabulary production and comprehension / baseline	None
Vismara et al. [34], 2012	Analysis of parent fidelity over time during treatment revealed significant increases from baseline to 12 weeks on the P-ESDM Fidelity Tool (0 no competent teaching to a score of 5 or extremely competent teaching). There were also increases on the Maternal Behaviour Rating Scale subscales of parent responsivity, parent affect, achievement oriented behaviours, and directive behaviour	Child Social Communication Behaviours (spontaneous or prompted verbalizations etc.), and the attention and initiation subscales of the Child Behaviour Rating Scale improved after the intervention	None
**Economic model**			
Bradford et al. [36] , 2014	None		The costs per consultation for the home telehealth program, the outpatient consultation, and the home visit were $294, $748, and $1214, respectively
**Cross-sectional & observational**			
Braverman et al [37], 2011	Using a 1- 5 scale, where 1 represents 'definitely yes,' the majority of participants responded '1' when asked about whether the consultation was useful 78% (46/59) and if they trusted the consultation 54.2% (32/59)	None	The three main types of help provided by email consultation was giving information about common diagnoses and treatment (89%), giving general information (59%), and giving recommendations about daily routine (30%). Half of the users engaged in short (one message) correspondence (35/70), 43% of the users (30/70) engaged in longer correspondence, 10% of the users (7/70) returned to the physician 1–7 months after the initial correspondence
Byczkowski et al. [38], 2014	62% of parents felt the communication with the	None	58% of parents sometimes used the portal instead of calling

	health professional was improved, 43% felt the quality of relationship with the health professional was improved. 81% of parents agreed the email function was very useful, 74% declared they received the information they needed, 71% reported receiving answers in a timely manner, 18% were sometimes concerned that emails/questions were sent to unintended recipients		their healthcare provider; 39% of parents used the portal to send emails to their health care professional
de Graaf et al. [39], 2013	89.9% of patients followed the advice of dermatologists and 98.4% would recommend the eHealth intervention to other parents<br>91.3% of parents perceived the eHealth intervention as useful and 92.2% found the eHealth intervention easy to use	None	
Hanlon-Dearman et al. [40], 2014	81% of participants reported being happy with their experience. All participants would recommend telehealth to another family	None	
Hopper et al. [41], 2011	100% reported satisfaction with the telegenetic concept for clinics, agreed that they had enough opportunity to ask questions. 50% found telemedicine was effective to see a geneticist. 25% felt that a face-to-face appointment was important for a first appointment with the clinical geneticist. 25% speculated that the effectiveness of telemedicine ‘depends on how a child deals with TV intrusion’, and a follow-up appointment with the geneticist in person was important. 88% agreed that telegenetics was an effective way of seeing a geneticist	None	

	for assessment.	
Lee et al. [42], 2010	Parents agreed that digital pictures of children's eye findings 1) will improve the quality of care from his/her doctor, 2) will help better understanding his/her disease condition, 3) could diagnose disease as accurately as an actual eye exam, and 4) overall, technology will improve the quality of medical care for my child. Parents did not agree that the potential benefits of sending these pictures electronically are worth the privacy risks, or that the diagnosis by far-away medical expert will make it easier to get the best possible health care for their child. Parents disagreed that the quality of care from diagnosis by far-away medical experts is better than the care from face-to-face medicine, and overall, that technology will make it harder for a patient and doctor to establish a good relationship	None
Mulgrew et al. [43], 2011	No difference between groups on overall parent satisfaction, satisfaction with the consulting healthcare provider, satisfaction with appointment availability, or in parents' comfort level in discussing health problems with provider	None
Nordfeldt et al. [44], 2010	Positive attitude of patients and parents towards the portal, finding it easy to use. Advantages of the portal included the availability of information when it is needed and the ability to search and find information quickly. Barriers for parents included the need for a	None

	password to access the portal		
Wade et al. [45], 2009	57.1% mothers (4/7) and 80% fathers (4/5) considered the videoconferencing easy to use overall. 71.4% mothers (5/7) and 60% fathers (3/5) considered the video conferencing helpful overall and 42.9% mothers (3/7) and 60% fathers (3/5) considered the videoconferencing helpful compared to face to face meetings	62.5% of teens (5/8) considered the videoconferencing easy to use overall. 87.5% of teens (7/8) considered the video conferencing helpful overall and 62.5% teens considered (5/8) the videoconferencing helpful compared to face to face meetings	None
**Interview studies**			
Barlott et al. [46], 2015	Key themes identified were the strength provided by a social support network, the potential benefit of community participation and the possibility that their experience of disability could change	None	
Britto et al. [47], 2013	Key themes related to benefits of using the portal including removing barriers to communication, providing a sense of control and providing reassurance	None	

[a]Health care professional outcomes are not reported in the table given the very limited studies with this outcome type.
