# Supplementary material for: Digital Clinical Communication for Families and Caregivers of Children or Young People With Short- or Long-Term Conditions: Rapid Review
Source: J Med Internet Res. 2018 Jan 5;20(1):e5. doi: 10.2196/jmir.7999 (PMC5775486; doi:10.2196/jmir.7999)
Supplement: Multimedia Appendix 5 [file jmir_v20i1e5_app5.pdf]

Multimedia Appendix 5. Complete results.

Author, Year, Study design	Results
Barlott et al. [46], 2015, Interview study	**Parent/carers outcomes:** Participation in the study provided caregivers with the possibility of a social support network, which gave them strength. Participation also showed caregivers the possibility of community participation and the possibility that their experience of disability could change, giving them optimism<br><br>**Health service outcomes:** In total, 50 messages were sent by SMS (6.25/participant) over a 3-month period: 20 were question messages (2.5/ participant; range, 0 to 12), and 30 were social interaction messages (3.75/ participant; range, 0 to 8)
Becker et al. [28], 2012, Pre/post intervention uncontrolled	**Patient outcomes:** The Eyberg Child Behaviour Inventory (assessing the frequency and severity of disruptive behaviours) reduced from baseline to 2 weeks for both the email and phone groups, with no differences between the two groups<br><br>**Parents/carers outcomes:** Similarly, there were no differences between the email and phone groups on the Oral Outcome Rating Scale, or the overall PAL (parent advice line) program satisfaction score
Bergmo et al. [10], 2009, RCT	**Patient Outcomes:** The objective Severity scoring of atopic dermatitis (AD) at 12 months was improved in both groups compared to that at baseline but there was no difference between the intervention and control groups<br><br>**Parent/carers outcomes:** There was an improvement in both groups regarding the number of skin care treatments per week performed by the parents at 12 months but no differences between the two groups were observed.<br>The intervention did not impact family costs at 12 months or loss of employment at 12 months<br><br>**Health services outcomes:** On average, the mean number of messages sent at 12 months was 8.3/participant.<br>There were fewer health care visits at 12 months in both groups compared with baseline but no difference between the two groups
Binford Hopf et al. [29], 2013, Pre/post intervention uncontrolled	**Parents/carers outcomes:**<br><u>After chat session:</u> 95.9% participants felt supported by the therapist in today's chat, 85.6% felt the advice and guidance they received from the therapist in today's chat was helpful, 9.6% felt the technical environment limit their ability to express yourself or to self-disclose in today's chat, 22.8% experienced technical problems (type of problem not reported).<br><br>There was an improvement on the Eating Disorder Symptom Impact Scale total score (P=.02). There was also improvement on the stigma (P=.02), and dependency (P=.05) subscales of the experience of Caregiving Inventory. There were no differences on the difficult behaviours (P=.30), negative symptoms (P=.44), problems with services (P=.33), effects on family (P=.06), need to backup (P=.59), loss (P=.55), positive personal experience (P=.58) or good aspects of relationship (P=.48) subscales
Bradford et al. [36] , 2014, Economic model	**Health services outcomes:** The costs per consultation for the home telehealth program, the outpatient consultation, and the home visit were $294, $748, and $1214 respectively
Bradford et al.[11], 2012, Non-randomised controlled study	**Parent/carer outcomes:** The HRQoL scores using the Life Threatening Illness-Family (QOLLTI-F) instrument improved in both groups compared to baseline but there were no differences between video-consultation and usual care

Braverman et al [37], 2011, Cross-sectional study	**Parent/carers outcomes:** Using a 1- 5 scale, where 1 represents ‘definitely yes,’ the majority of participants responded ‘1’ when asked about whether the consultation was useful 78% (46/59) and if they trusted the consultation 54.2% (32/59).<br><br>**Health service outcomes:** The three main types of help provided by email consultation was giving information about common diagnoses and treatment (89%), giving general information (59%), and giving recommendations about daily routine (30%). Half of the users engaged in short (one message) correspondence (35/70), 43% of the users (30/70) engaged in longer correspondence (i.e. two or more email messages on the same topic from the patient), 10% of the users (7/70) returned to the physician 1–7 months after the initial correspondence
Britto et al. [47], 2013, Interview study	**Parent/carers outcomes:** Parents reported benefits of using the portal including removing barriers to communication, providing a sense of control and providing reassurance
Byczkowski et al. [38], 2014, Cross-sectional Study	**Parent/carer outcomes:** 62% of parents felt the communication with health professional was improved, 43% felt the quality of relationship with health professional was improved.<br>81% of parents agreed the email function was very useful, 74% declared they received the information they needed, 71% reported they reported receiving answers in a timely manner, 18% were sometimes concerned that emails/questions were sent to unintended recipients.<br><br>**Health service outcomes:** The proportion of parents that used the portal instead of calling their healthcare provider sometimes was 58% and usually/ always 6%; 39% of parents used the portal to send emails to their health care professional
de Graaf et al. [39], 2013, Cross-sectional Study	**Parent/carer outcomes:** After using the web-based eHealth intervention, 89.9% of patients followed the advice of dermatologists and 98.4% would recommend the eHealth intervention to other parents<br>91.3% of parents perceived the eHealth intervention as useful and 92.2% found the eHealth intervention easy to use
Epstein et al. [30], 2015, Pre/post intervention uncontrolled with interview study	**Parent/carer outcomes:** The video-conferencing system was rated as ‘good’ or ‘excellent’ by 100% of parents for ease of updates, and 78% for video and audio quality. More than 90% of parents perceived the intervention to be reliable for updates in the NICU.<br>At 5 days, there was no difference on parents’ understanding survey scores compared to baseline.<br><br>**Healthcare professional outcomes:** More than 90% of providers perceived the intervention to be reliable for updates in the NICU. 94% of providers rated the ease of using video conferencing as excellent or good
Grover et al. [19], 2011, RCT	**Parents/carers outcomes:** Of the 33 carers randomised to the web-intervention (overcoming anorexia online), 78.8% requested email support as part of the package and 21.2% preferred telephone support
Gulmans et al. [31], 2012, Pre/post intervention uncontrolled	**Parents/carers outcomes:** The web-based system for parent-professional communication was evaluated using a survey of 20 parents: 70% responded that the system considerably contributed to sufficient contact and 65% responded it considerably contributed to timely information exchange; 65% considered that the system considerably contributed to better accessibility for consultation. The consistency of information was rated using several items and rated as positive in 45% of responses, 10-25% did not know and 25-35% had a negative experience. Regarding the parents’ messenger/coordinator role, 45% had a positive experience, 10-25 % did not know, and 25-35% negative experience

Gund et al. [20], 2013, RCT	**Parent/ carer outcomes:** Web-based system: 100% of parents found the experience extensive, 100% found the application easy to use, 83% found the application to be good or very good, 50 % found that webpage help feel more confident in the care of child at home, 33% found that the application reduced the need of home visits.<br>Skype: 100% of families found Skype easy to use, 100% were satisfied with the video calls, 87.5% found easy to communicate with the nurse by video call, 75% found the answers or instructions received during the video calls useful,<br>50% found video calls less stressful than home visits, 100 % found that webpage help feel more confident in the care of child at home, 75% found that the application reduced the need of home visits.<br><br>**Healthcare professional outcomes:** Most nurses were motivated to use the information and communication technology but one nurse reported seeing the study as a threat and avoided using the intervention
Hanberger et al. [21], 2013, RCT	**Patient Outcomes:** The HBA1c level was 6.8% at baseline and 6.7% at 12 months in both groups (no significant differences).<br>Although actual figures were not provided, no differences were reported between the intervention and control groups for severe self-reported hypoglycaemia, self-controlled blood glucose, HRQoL, satisfaction/acceptance, and empowerment.<br><br>**Parent/ carer outcomes:** Although actual figures were not provided, no differences were reported between the intervention and control groups for HRQoL, satisfaction/acceptance, and empowerment
Haney et al. [32], 2012, Pre/post intervention uncontrolled	**Parent/ carer outcomes:** The Pediatric Quality of Life subscales family impact and healthcare satisfaction were not statistically improved at 12 weeks compared to baseline
Hanlon-Dearman et al. [40], 2014, Cross-sectional Study	**Parent/ carer outcomes:** 81% of participants reported being happy with their experience of telehealth for diagnosis and follow-up support. All participants would recommend telehealth to another family
Hopper et al. [41], 2011, Observational study (Survey of carers and consultation content)	**Parent/ carer outcomes:** 100% respondents reported that they were satisfied with the telegenetic concept for clinics, agreed that they had enough opportunity to ask questions at the telemedicine and face-to-face appointment<br>50% respondents agreed that telemedicine was an effective medium to see a geneticist for the purpose of trying to ascertain a genetic diagnosis for their child.<br>25% felt that a face-to-face appointment was important for a first appointment with the clinical geneticist *"because cameras don't show everything."*<br>25% speculated that the effectiveness of telemedicine 'depends on how a child deals with TV intrusion', and a follow-up appointment with the geneticist in person was important.<br>All respondents were either satisfied or very satisfied with the telemedicine experience. Respondents were asked their opinion about improving the provision of telegenetics; however, no suggestions were made. All carers were given enough time to ask questions at both appointments and were happy with the information given at the telegenetic clinic. One parent commented that they *"did not know what kind of questions to ask"* in the telemedicine appointment. The majority of participants (88%) agreed that telegenetics was an effective way of seeing a geneticist for assessment.<br><br>**Health service outcomes:** The measurements taken by the genetic counsellor and the geneticist varied slightly but not sufficiently to affect the assessment and alter a diagnosis

Lee et al. [42], 2010, Cross-sectional Study	**Parent/ carer outcomes:** Using a 5-point Likert scale (where 1 is strongly disagree, 3 is neutral and 5 is strongly agree)<br><br>On average parents agreed with the following statements: (i) Digital pictures of my child's eye findings will improve the quality of care from his/her doctor, (ii) Digital pictures of my child's eye findings will help me better understanding his/her disease condition, (iii) Digital pictures of my child's eye findings could diagnose disease as accurately as an actual eye exam, (iv) Digital pictures of my child's retinopathy should be included in the permanent medical record, (v) Overall, technology will improve the quality of medical care for my child, (vi) It is essential to meet face-to-face with my child's doctor.<br><br>On average parents did not agree or disagree with the following statements: (i) Digital cameras and computers are reliable, (ii) The potential benefits of sending these pictures electronically are worth the privacy risks, (iii) Diagnosis by far-away medical expert will make it easier to get the best possible health care for my child, (iv) The quality of care from diagnosis by far-away medical experts is as good as the care from face-to-face medicine, (v) I could not trust a doctor that I did not personally meet.<br><br>On average parents disagreed with the following statements: (i) The quality of care from diagnosis by far-away medical experts is better than the care from face-to-face medicine, (ii) I am worried sending these pictures electronically will create risks for the privacy of my child's medical information, (iii) I am worried that a computer may not send the pictures correctly, (iv) Overall, technology will make it harder for a patient and doctor to establish a good relationship
Looman et al. [22], 2015, RCT	**Health service outcomes:** There was no statistically significant change between baseline and 24 months after, and no differences between the three groups regarding the following items: global rating of health care, family-centred care, getting needed care, getting care quickly, provider communication, and help discrepancy (help needed - help received).<br><br>Scores for personal doctor rating and adequacy of care coordination were rated higher in both intervention groups (telephone and video) compared to the control group (P=.001 and P=.026, respectively)
Mulgrew et al. [43], 2011, Cross- sectional Study	**Parent/ carer outcomes:** There was no statistically significant difference between the intervention and control groups on overall parent satisfaction, satisfaction with the consulting healthcare provider, satisfaction with appointment availability, or in parents' comfort level in discussing health problems with provider.<br>When asked whether the provider explained things about the child's health in a way that is easy to understand, the rating of visits was lower for telehealth visits compared to face-to-face visits (P=.01)
Nordfeldt et al. [44], 2010, Cross-sectional Study	**Patient outcomes:** Patients had a positive attitude towards the portal, finding it easy to use. Advantages of the portal included the availability of information when it is needed and the ability to search and find information quickly. One patient reported that a more functional chatroom was needed.<br><br>**Parent/carer outcomes:** Parents had a positive attitude towards the portal, finding it easy to use. Advantages of the portal reported included peer to peer communication, the availability of information when it is needed and the ability to search for information. Barriers included the need for a password to access the portal

Petranovich et al. [23], 2015, RCT	No outcomes related to the communication component of the program were reported
Scharer et al. [24], 2009, RCT	**Parent/ carer outcomes:** Mothers in the chat room intervention group had to be active in seeking support (e.g. by signing into the chat room to access the nurse). In the telephone intervention group both active and passive mothers received consistent support
Van Os-Medendorp et al. [25], 2012, RCT	**Patient outcomes:** There was no significant differences between the intervention and control groups over time for the Infants' Dermatitis Quality of Life Index (IDQOL). The intensity of itching was not statistically different between the groups. The severity of AD differed significantly over time between the two groups (P = .04). However, the differences between the groups at each time point were not significantly different.<br>**Health service outcomes:** Total costs (direct + indirect) at 12 months were €3378 for patients using individualised e-health and €3972 for patients with usual care (face-to-face care)
Vismara et al. [33], 2013, Pre/post intervention uncontrolled	**Patient outcomes:** Child behaviour: the rates of vocalizations and joint attention initiations increased from baseline to follow-up<br>The means of vocabulary production and comprehension also increased from baseline to follow-up.<br><br>**Parent/ carer outcomes:**<br>All eight parents agreed or strongly agreed that the website was easy to use, that there was enough information given to meet learning needs, that they were able to use the telehealth intervention to increase their child's language, play, and social engagement skills, they felt well supported by the intervention and would recommend it to other families. Only one person did not agree or strongly agree that the video conferencing sessions and online modules were the most helpful aspects of the intervention, or would feel confident teaching other caretakers to use the intervention with their child.<br><br>The Maternal Behaviour Rating Scale (designed to assess the quality of maternal interactive behavior with children with learning difficulties) score increased from baseline to follow-up (no p value reported)
Vismara et al. [34], 2012, Pre/post intervention uncontrolled	**Patient outcomes:** Child Social Communication Behaviours (spontaneous or prompted verbalizations etc.), and the attention and initiation subscales of the Child Behaviour Rating Scale improved after the intervention<br><br>**Parent/carer outcomes:** 88.9% of parents (8/9) expressed initial concerns about whether telehealth delivery would provide enough support to change behaviour and the logistics of using the software program. When asked whether initial concerns were addressed by the end of the study, these parents felt reassured and perceived the distance coaching as informative and as valuable as live in-home or center-based sessions delivered by professionals. All parents described some degree of frustration when using the video conferencing program, such as the Internet connection freezing in mid-conversation or the audio or web-camera not working when first connecting with the therapist.<br>Analysis of parent fidelity over time during treatment revealed significant increases from baseline to 12 weeks on the P-ESDM Fidelity Tool (0 no competent teaching to a score of 5 or extremely competent teaching). There were also increases on the Maternal Behaviour Rating Scale subscales of parent responsivity, parent affect, achievement oriented behaviours, and directive behaviour
Wade et al. [26], 2014, RCT	**Parent/ carer outcomes:** Both the CAPS intervention and IRC intervention were associated with a reduction in caregiver distress. Participants who completed 5 or more CAPS sessions reported a greater reduction in depression that participants in the IRC intervention group

Wade et al. [35], 2009, Pre/post intervention uncontrolled	**Parent/ carer outcomes:** 100% of parents found Skype videoconferences to be helpful
Wade et al. [27], 2012, RCT	**Parent/ carer outcomes:** No results were provided regarding the helpfulness of skype videoconferences
Wade et al. [45], 2009, Cross – sectional study	**Patient outcomes:** 62.5% of teens (5/8) considered the videoconferencing easy to use overall. 87.5% of teens (7/8) considered the video conferencing helpful overall and 62.5% teens considered (5/8) the videoconferencing helpful compared to face to face meetings.<br><br>**Parent/ carer outcomes:** 57.1% mothers (4/7) and 80% fathers (4/5) considered the videoconferencing easy to use overall. 71.4% mothers (5/7) and 60% fathers (3/5) considered the video conferencing helpful overall and 42.9% mothers (3/7) and 60% fathers (3/5) considered the videoconferencing helpful compared to face to face meetings
